# Editorial Department

**Published:** 1840

**Authors:** 


### EDITORIAL DEPARTMENT.

In establishing this department we did not propose to sit, like Diogenes in his tub, solitary and snarling; but would rather invite in all the friends, who are disposed to enter with their paragraphs of new and insulated facts or passing suggestions and remarks. We have even thought of labelling it with the expressive anatomical term

CONGLOMERATION; or, exchanging the noun for the verb—(it is always easy to substitute the *word* for the *substance*)—adopt as our motto *Conglomeramus*.

Few of the days of an observing physician ever pass, without presenting him with particular facts, or suggesting doubts on apparently established points, or prompting inquiries on those which are unsettled: It is our wish to make this department of the Journal, a place of deposit for all such *semina*, and we believe, at least hope, that he who sows will find, that he has not cast his seed on barren ground. Such acts of professional husbandry, would improve his mental condition, while posterity would reap, in many instances an hundred fold. The field of science is illimitable, and has varieties of soil adapted to the reception and growth of every kind of germ. We say, then, to our friends—old and young, the ripe and the unripe in science, take up your pens, and put us in possession of your observations. They will be wafted far and wide over the west, as feathered seeds float upon the winds, to distribute equally the bounties of the vegetable kingdom. Interchange of thought is the great secret of improvement; and a community of labor is indispensable to success, in all the sciences which rest upon observation and experiment. We are happy to know that our colleagues of the Institute intend to contribute to *this* as well as the other departments, of the Journal. The contributions of each will be indicated by the initial of his signature. D.

